# Temporal and spatial evolution characteristics and decoupling trend of Chinese agricultural carbon emission efficiency

**DOI:** 10.1371/journal.pone.0311562

**Published:** 2024-10-10

**Authors:** Xiaochun Zhao, Danjie Yang, Xin Duan

**Affiliations:** School of Management, Anhui University, Hefei, China; Zhongnan University of Economics and Law, CHINA

## Abstract

This study investigates the spatio-temporal evolution of agricultural carbon emission efficiency (ACEE) in China and its relationship with agricultural economic growth (AEG). The results indicate several findings: Firstly, between 2012 and 2021, China’s agricultural carbon emission efficiency exhibited an upward trend, with the mean value increased from 0.349 to 0.807. Furthermore, the distribution pattern shifted from a dispersed, point-like distribution to an aggregated and continuous distribution. Secondly, the average agricultural carbon emission efficiency in China following a decreasing order: South China, Northwest China, Southwest China, East China, North China, Central China and Northeast China. Thirdly, the relationship between agricultural carbon emission efficiency and the agricultural economy in China has transitioned from weak decoupling to negative decoupling. Based on these findings, this study proposes some recommendations to enhance agricultural carbon emission efficiency and promote its decoupling from agricultural economic growth. These recommendations aim to achieve low-carbon and high-efficiency development of agriculture.

## 1 Introduction

Global warming, resulting from massive greenhouse gas (GHG) emissions, poses a significant threat to human development. The reduction of carbon emissions has become an issue of utmost importance for many countries. According to a report released by the Food and Agriculture Organization of the United Nations (FAO) at the COP26 Climate Summit on November 8, 2021, global greenhouse gas emissions from agriculture and food production have increased by 17% over the past 30 years. Carbon emissions from agricultural activities, such as farming and crop cultivation, have become major contributors to global greenhouse gas levels [1]. Moreover, global climate change, caused by greenhouse gas emissions, pose a threat on agriculture development [2]. In light of this threat, urgent action is required to reduce carbon emissions. However, developing countries face particular challenges in implementing carbon reduction strategies in agriculture due to limited technology, knowledge and financial resources. China, as the largest developing country, is also the largest emitter of carbon. China’s agricultural production alone accounts for 16% to 17% of the global greenhouse gases generated by agriculture [3]. Thus, it is imperative to find effective ways to reduce agricultural carbon emissions while maintaining stable agricultural economic growth. Although existing studies have examined agricultural carbon emissions, their spatial and temporal distribution, agricultural landscapes and agricultural carbon emission efficiency [4–6], there has been limited analysis of the relationship between agricultural carbon emission efficiency and agricultural economic development. It is crucial to explore effective ways to improve the efficiency of agricultural carbon emissions while promoting the development of agriculture. Therefore, this study aims to assess China’s agricultural carbon emission efficiency and further analyze its decoupling relationship with agricultural economic growth. This paper not only provides insights into promoting sustainable agriculture in China, but also offers invaluable inspiration for other developing country to develop low-carbon agriculture.

The subsequent sections of this study are structured as follows: The second part reviews relevant literature on agricultural carbon emission efficiency, the third part introduces the research methodology and data sources, the fourth part analyzes the agricultural carbon emission efficiency and its relationship with the agricultural economy in China, and the fifth part summarizes the research results and puts forward targeted suggestions, and also discusses the limitations of this paper.

## 2 Literature review

At present, the research about agricultural carbon emissions can generally be categorized into three areas: measuring agricultural carbon emission efficiency, identifying factors that influence agricultural carbon emission efficiency and analyzing the spatial and temporal evolution as well as the spatial effects of agricultural carbon emissions.

First of all, scholars assess agricultural carbon emissions using various evaluation methods, including input-output analysis, life cycle approach and Data Envelopment Analysis (DEA) [7–9]. For instance, Elahi et al. used the Slacks-Based Measure model, modified gravity model and social network analysis to evaluate carbon emission efficiency in food production, determine spatial correlations, categorize network clusters, and identify influential drivers [10]. Wang et al. employed DEA and the Malmquist index model to measure the carbon emission efficiency of agriculture from both static and dynamic perspectives [11].

Secondly, researchers have explored the factors influencing agricultural carbon emission efficiency using various methods. For example, Shi et al. applied the Kuznets curve and panel regression model to examine the relationship between agricultural carbon emission efficiency and the level of agricultural economy [12]. Additionally, scholars utilized the Logarithmic Mean Divisia Index (LMDI) model to decompose the factors affecting agricultural carbon emission efficiency, finding that agricultural industrial structure, economic development, and urbanization positively impact agricultural carbon emission efficiency [13]. Conversely, government financial expenditure, agricultural planting structure, and agricultural disasters have negative impacts [14]. Furthermore, digital technology also plays an important role in improving carbon emission efficiency [15].

Finally, regarding the spatio-temporal evolution of agricultural carbon emission efficiency, Hou et al. used the Dagum Gini coefficient, GeoDetector model and Panel Geographically-Temporally Weighted Regression model to examine regional differences, sources of reduction effects in pollution and carbon emissions, identify dominant factors, and investigate their spatial-temporal heterogeneity impacts [16]. Zhang et al. used the Kernel density model to analyze the internal dynamic evolution of agricultural carbon emission efficiency, discovering a certain degree of spatial aggregation in northern and southern China [17]. The similarities in production conditions in neighboring regions may lead to spatial correlation in carbon-emission efficiency [18]. Tang et al. employed a spatial Markov chain study, concluding that agricultural carbon emission efficiency exhibits club convergence. Therefore, the exploration of agricultural carbon emission efficiency should consider both the regional situation and correlations with surrounding regions [19].

In summary, the aforementioned research on agricultural carbon emission efficiency lays the foundation for this paper. However, most of the literature focus on the impacts of agricultural production efficiency, financial agglomeration, renewable energy consumption and agricultural economic growth on agricultural carbon emissions, as well as the decoupling relationship between agricultural carbon emissions, and food production and the decoupling effect between agricultural carbon emission intensity and agricultural development [20–24]. There is a lack of research on the decoupling relationship between carbon emission efficiency and agricultural economic growth. The advantage of carbon emission efficiency is that it not only focuses on the total amount of carbon emissions, but also takes into account economic output, energy consumption and other factors, which helps to reveal the impact of agricultural carbon emissions on agricultural economic development more accurately.

Therefore, the innovation of this paper lies in using the Super Slacks-Based Measurement (Super-SBM) model to evaluate the agricultural carbon emission efficiency of 30 provinces in China from 2012 to 2021. Based on this evaluation, the Tapio decoupling model is employed to explore the decoupling relationship between China’s agricultural carbon emission efficiency and agricultural economic growth. Through comparative the differences among China’s seven major regions, this paper aims to reveal the spatio-temporal evolution characteristics of China’s agricultural carbon emission efficiency and the degree of decoupling between it and agricultural economic growth. Ultimately, this research endeavors to provide insights for China to achieve a green and low-carbon transformation in agriculture.

## 3 Research design

### 3.1 Super-SBM model

The traditional DEA model does not account for the relaxation of inputs and outputs during efficiency evaluation. Additionally, it excludes undesirable outputs from its measurement scope, leading to biased efficiency assessments due to radial and angular choices. To address these issues, Tone proposed a non-radial and non-angular SBM model in 2001 [25]. Nevertheless, the SBM model may produce multiple decision-making units (DMUs) with efficiency values equal to one, complicating the comparison of efficiency levels among effective DMUs and impacting decision-making accuracy. To resolve this problem, Tone further refined the SBM model and introduced the Super-SBM model with non-expected outputs in 2002 [26]. This model integrates the expected outputs into the framework of the existing model, taking into account the relationships between inputs, outputs, and undesired outputs. The Super-SBM model not only allows for the detailed decomposition of decision-making units, thereby enhancing the practical applicability of the model, but also mitigates result biases caused by radial direction and angular choices. Consequently, this study employs the Super-SBM model to evaluate the agricultural carbon emission efficiency. The corresponding formula for the model can be found in formula (1).


$$\varphi =min\frac{\frac{1}{m}\sum_{i=1}^{m}\left(\frac{\bar{x}}{x_{ik}}\right)}{\frac{1}{r_{1}+r_{2}}\times \left(\sum_{s=1}^{r_{1}}\frac{\bar{y^{d}}}{y_{sk}^{d}}+\sum_{q=1}^{r_{2}}\frac{\bar{y^{u}}}{y_{qk}^{u}}\right)}$$

x¯≫∑j=1,≠knxijσji=1,2,...,myd¯≪∑j=1,≠knysjdσjs=1,2,...,r1yu¯≫∑j=1,≠knyquσjq=1,2,...,r2σj≥0j=1,2,...,nx¯≫xikj=1,2,...,myd¯≪yskds=1,2,...,r1yu¯≫yquu=1,2,...,r2 (1)

In formula (1), *φ* represents the target efficiency value, which corresponds to the carbon emission efficiency of China’s agriculture; this study considers the efficiency values of *n* DMUs. Each DMU is composed of an input matrix *m*, an expected output matrix *r*_*1*_, and an undesired output matrix *r*_*2*_; *x*, $y_{s}^{d}$ and $y_{q}^{u}$ represent inputs, desired outputs and undesired outputs, respectively; $\bar{x}$, *y*^*d*^ and *y*^*u*^ represent input relaxation, desired output relaxation and undesired output relaxation, respectively.

### 3.2 Designing indicator system for agricultural carbon emission efficiency

In order to evaluate agricultural carbon emission efficiency scientifically, it is necessary to design an indicator system for agricultural carbon emission efficiency (see Table 1).

**Table 1:** Evaluation indicator system of agricultural carbon emission efficiency.

Indicators	Primary indicators	Secondary indicators	Unit
Input indicators	Labour force	Employees in the primary industry	%
	Land	Crop sown area	%
	Water resource	Effective irrigated area	%
	Agricultural materials	Fertilizer	10,000 tons/thousand hectares
		Pesticide	10,000 tons/thousand hectares
		Agricultural film	10,000 tons/thousand hectares
		Agricultural machinery power	10,000 kW/thousand hectares
Output indicators	Desired output	Gross value of agricultural output	Billion/thousand hectares
	Undesired output	Agricultural CO_2_ emissions	10,000 tons/thousand hectares

Firstly, regarding input indicators, referring to the study of Han et al. [27]. There are four items to consider: (1) Labour, labor input is represented by the ratio of the number of people employed in the primary industry at the end of the year in each province to the total number of people employed at the end of the year in each province. (2) Land, the amount of land input for agriculture is represented by the ratio of the sown area of crops in the calendar year to the total area of the province. This ratio accounts for differences in the replanting index of each province and the impact of fallow land in some areas. (3) Water resources, water, an essential resource for agricultural production, is quantified by the ratio of the effective irrigated area of agriculture to the sown area of crops in a calendar year.

(4) Agricultural materials, referring existing literature and general agriculture production [28], four variables are used to represent agricultural materials inputs: fertilizer, pesticide, agricultural film and agricultural machinery power. Fertilizer is represented by the ratio of the total fertilizer use to the sown area of crops. Pesticide is represented by the ratio of the total pesticide use to the sown area of crops. Agricultural film is represented by the ratio of the total film use to the sown area of crops. Power of agricultural machinery is represented by the ratio of the total rated power of agricultural machinery in agricultural activities to the sown area of crops in each province.

Secondly, regarding output indicators. Referring to the research of Rehman A et al. [29], this study uses the ratio of the annual gross value of agricultural output to the area sown in agriculture for each province to express the desired output of carbon emission efficiency of agriculture. For the undesired output indicator, the undesired output is characterized by the ratio of agricultural CO_2_ emissions to the sown area of crops in each province, as guided by the Guidelines for National Greenhouse Gas Emission Inventories published by the Oak Ridge National Laboratory (ORNL), the United Nations Intergovernmental Panel on Climate Change (IPCC), and the measured coefficients from the College of Biology and Technology of China Agricultural University (CBTCAU).

Thirdly, this study calculates only the greenhouse gases produced by agricultural production, including CO_2_ and N_2_O from farmland soil, agricultural irrigation, pesticides, diesel fuel, and other sources, which are converted into standard carbon. The formula for calculating total agricultural carbon emissions is as follows:

$$CT=\sum N_{\text{i}}E_{i}$$ (2)

In formula (2), *CT* represents the total carbon emissions of agriculture; *N*_*i*_ represents the amount of carbon source *i*; and *E*_*i*_ represents the emission coefficient of carbon source *i* (see Table 2).

**Table 2:** Agricultural carbon emissions sources and coefficients.

Carbon source	coefficient
Fertilizer	0.89kg C/kg
Pesticide	4.93kg C/kg
Agricultural film	5.18kg C/kg
Diesel	0.59kg C/kg
Irrigation	266.48kg C/hm^2^
Plough	312. 60kg C/hm^2^

### 3.3 Tapio model

In 2005, the Organization for Economic Co-operation and Development (OECD) first proposed the decoupling theory, which indicated that there is no linear relationship between economic growth and environmental pollution. However, the OECD model can be influenced by the selected time period, making comparative analyses difficult. To address this, Tapio P et al. proposed the Tapio decoupling model in 2005 [30]. This model introduces critical values of 0, 0.8, and 1.2, integrating both relative and absolute quantities, thus being less affected by the selected time base period and leading to more stable measurement results [31]. This study adopts the Tapio model to analyze the decoupling relationship between agricultural carbon emission efficiency and the agricultural economy. The formula for the decoupling index is shown in formula (3).

$${\text{e}}_{\left(CEE,GDP\right)}=\frac{\Delta CEE}{\Delta GDP}=\frac{\left(CEE_{t}-CEE_{t-1}\right)/CEE_{t-1}}{\left(GDP_{t}-GDP_{t-1}\right)/GDP_{t-1}}$$ (3)

*e*_(*CEE*,*GDP*)_ represents the index of the decoupling of agricultural carbon efficiency from economic development; Δ*CEE* represents the rate of change in the carbon emission efficiency of Chinese agriculture; Δ*GDP* represents the rate of change of growth in agricultural economic development, and the data is derived from the gross value of agricultural production in each province.

Based on the Tapio decoupling model [32], which categorizes decoupling into eight types, this study uses the criteria to classify the decoupling status of agricultural carbon emission efficiency and the agricultural economy (see Table 3).

**Table 3:** Criteria for classifying the decoupling of agricultural carbon emission efficiency and agricultural economy.

Types of decoupling	Decoupled state	ΔCEE	ΔGDP	e
Negative decoupling	Weak-negative decoupling (N)	<0	<0	0≤e<0.80
	Strong-negative decoupling (I)	>0	<0	e<0
	Expansion negative decoupling (E)	>0	>0	e>1.20
Decoupling	Recession decoupling (R)	<0	<0	e>1.20
	Strong decoupling (S)	<0	>0	e<0
	Weak decoupling (W)	>0	>0	0≤e<0.80
Connect	Recession Connection (G)	<0	<0	0.80≤e≤1.20
	Expansion Connection (D)	>0	>0	0.80≤e≤1.20

### 3.4 Data sources

As the yearbook for 2023 has not yet been released, the analysis focuses on data from 2012 to 2021 for 30 provinces in China (excluding Hong Kong, Macao, Taiwan, and Tibet). The data were obtained from the China Statistical Yearbook (2013–2022), China Agricultural Yearbook (2013–2022), and China Rural Statistical Yearbook (2013–2022).

## 4 Analysis results

### 4.1 Evaluation results of agricultural carbon emission efficiency

According to The Fourth National Assessment Report on Climate Change, jointly compiled in 2022 by the Ministry of Science and Technology of China, China Meteorological Administration, Chinese Academy of Sciences, and Chinese Academy of Engineering and the study of Sang et al., China was divided into seven regions: North China, Northeast China, East China, Central China, South China, Southwest China, and Northwest China [33]. The Super-SBM model with undesired outputs was used to calculate agricultural carbon emission efficiency for each region in China from 2012 to 2021. The results for all 30 provinces are presented in Table 4. Referring to the study of Ray S [34], agricultural carbon emission efficiency was classified into five classes: 0–0.300 (low level), 0.301–0.400 (lower level), 0.401–0.500 (medium level), 0.501–0.800 (higher level), and more than 0.801 (high level).

**Table 4:** Carbon emission efficiency of China’s agriculture from 2012 to 2021.

Region	Province	2012	2013	2014	2015	2016	2017	2018	2019	2020	2021	Averages
North China	Beijing	0.574	0.671	0.687	0.755	0.815	0.840	0.843	1.018	0.899	1.482	0.858
	Tianjin	0.367	0.417	0.435	0.460	0.475	0.565	0.673	0.722	0.862	1.170	0.615
	Hebei	0.269	0.294	0.285	0.280	0.298	0.325	0.375	0.392	0.457	0.501	0.348
	Shanxi	0.246	0.265	0.273	0.270	0.290	0.322	0.341	0.359	0.415	0.477	0.326
	Inner Mongoria	0.300	0.325	0.325	0.312	0.304	0.289	0.315	0.334	0.367	0.385	0.326
	Averages	0.351	0.394	0.401	0.415	0.436	0.468	0.509	0.565	0.600	0.803	0.494
Northeast China	Liaoning	0.295	0.317	0.318	0.369	0.348	0.359	0.393	0.439	0.486	0.533	0.386
	Jilin	0.221	0.231	0.228	0.218	0.256	0.172	0.193	0.198	0.241	0.257	0.222
	Heilongjiang	0.264	0.321	0.339	0.327	0.326	0.356	0.374	0.412	0.440	0.461	0.362
	Averages	0.260	0.290	0.295	0.305	0.310	0.295	0.320	0.350	0.389	0.417	0.323
Eastern China	Shanghai	0.766	0.814	0.764	0.721	0.643	0.684	1.010	0.924	0.884	1.071	0.828
	Jiangsu	0.306	0.324	0.340	0.378	0.380	0.397	0.401	0.420	0.457	0.511	0.391
	Zhejiang	0.365	0.399	0.425	0.447	0.455	0.523	0.554	1.056	0.649	0.701	0.557
	Anhui	0.179	0.185	0.194	0.197	0.211	0.226	0.227	0.240	0.286	0.321	0.227
	Fujian	0.463	0.494	0.553	0.589	0.674	0.721	0.792	0.871	0.921	1.083	0.716
	Jiangxi	0.203	0.243	0.258	0.295	0.317	0.333	0.364	0.403	0.437	0.466	0.332
	Shandong	0.240	0.274	0.474	0.295	0.306	0.316	0.345	0.376	0.405	0.470	0.350
	Averages	0.360	0.390	0.430	0.417	0.426	0.457	0.527	0.613	0.577	0.661	0.486
Central China	Henan	0.245	0.257	0.277	0.289	0.304	0.325	0.364	0.404	0.473	0.517	0.345
	Hubei	0.320	0.337	0.341	0.343	0.370	0.398	0.420	0.470	0.511	0.577	0.409
	Hunan	0.293	0.284	0.288	0.288	0.308	0.323	0.339	0.394	0.460	0.501	0.348
	Averages	0.277	0.301	0.331	0.325	0.336	0.355	0.384	0.416	0.469	0.513	0.371
Southern China	Guangdong	0.458	0.505	0.529	0.560	0.641	0.673	0.752	0.871	0.943	1.112	0.704
	Guangxi	0.367	0.387	0.407	0.422	0.456	0.504	0.546	0.626	0.660	0.763	0.514
	Hainan	0.418	0.427	0.478	0.506	0.598	0.621	0.683	0.770	0.836	1.790	0.713
	Averages	0.415	0.440	0.471	0.496	0.565	0.599	0.660	0.756	0.813	1.222	0.644
Southwest China	Chongqing	0.336	0.362	0.391	0.407	0.478	0.497	0.551	0.608	0.712	0.808	0.515
	Sichuan	0.415	0.428	0.450	0.478	0.538	0.581	0.610	0.652	0.707	0.784	0.564
	Guizhou	0.242	0.284	0.360	0.468	0.520	0.581	0.655	0.772	0.865	2.223	0.697
	Yunnan	0.268	0.304	0.323	0.321	0.334	0.349	0.422	0.522	0.570	0.687	0.410
	Averages	0.315	0.345	0.381	0.418	0.468	0.502	0.560	0.639	0.714	1.126	0.547
Northwest China	Shaanxi	0.473	0.533	0.583	0.584	0.628	0.660	0.707	0.781	0.916	1.087	0.695
	Gansu	0.205	0.219	0.225	0.236	0.263	0.306	0.346	0.393	0.429	0.533	0.316
	Qinghai	0.500	0.603	0.610	0.590	0.631	0.654	0.695	0.791	0.882	1.252	0.721
	Ningxia	0.263	0.298	0.316	0.357	0.380	0.395	0.448	0.438	0.527	0.545	0.397
	Xinjiang	0.623	0.626	0.574	0.599	0.601	0.635	0.703	0.730	0.827	1.148	0.706
	Averages	0.413	0.456	0.462	0.473	0.501	0.530	0.580	0.626	0.716	0.913	0.567
National average		0.349	0.381	0.402	0.412	0.438	0.465	0.514	0.579	0.617	0.807	0.496

Firstly, Table 4 shows that China’s average carbon emission efficiency increased from 0.349 in 2012 to 0.807 in 2021, demonstrating an improvement of 0.458. This consistent upward trend signifies significant progress in reducing agricultural carbon emissions and transitioning towards a more sustainable and low-carbon agriculture. Factors contributing to the achievement include China’s steadfast implementation of policies aimed at carbon sequestration and emission reduction, increased investment in agricultural environmental management, advancements in agricultural science and technology, and the promotion of low-carbon agricultural practices.

Secondly, at the provincial level, Table 4 reveals that Beijing and Shanghai have achieved a high level of average agricultural carbon emission efficiency. Additionally, Qinghai, Fujian, Hainan, Xinjiang, Guangdong, Guizhou, Shaanxi, and Tianjin also exhibit higher level of efficiency in agricultural carbon emissions. Sichuan, Zhejiang, Chongqing, Guangxi, Yunnan, and Hubei demonstrate a medium level of agricultural carbon emission efficiency. On the other hand, Ningxia, Jiangsu, Liaoning, Heilongjiang, Shandong, Hunan, Hebei, Henan, Jiangxi, Shanxi, Inner Mongolia, and Gansu exhibit lower level of agricultural carbon emissions efficiency. Anhui and Jilin, despite being major grain-producing provinces, present low level of efficiency due to high input and carbon emissions per unit of production, as well as low output per unit of production. What’s more, in 2012, only 3 provinces had higher agricultural carbon emission efficiency, but by 2021, there were already 10 provinces with agricultural carbon efficiency greater than 1, and 23 provinces with a relatively high level of agricultural carbon emission efficiency. This indicates a significant improvement in China’s agricultural carbon emission efficiency, transitioning from sporadic distribution to aggregated distribution. Considering that China is a vast country with diverse terrain and climate, it is important to note that agricultural production methods are influenced by factors such arable land availability, labor availability, level of agricultural technology development, natural resources, and industrial structure. These factors, in turn, affect the process of agricultural modernization and low-carbonization in each province. Therefore, it is crucial for each region to formulate a low-carbon agricultural development strategy based on local conditions. Overall, these findings highlight China’s progress in improving agricultural carbon emission efficiency and emphasize the importance of continued efforts in promoting sustainable and low-carbon agricultural practices.

Upon examining the data presented in Table 4, it is evident that there is significant regional variation in the efficiency of agricultural carbon emissions across different regions in China. Generally, the southern and northwestern regions of China exhibit higher levels of agricultural carbon emission efficiency compared to central China. The provinces with higher overall agricultural carbon emission efficiency are primarily located in the eastern and southern coastal regions, including Beijing, Shanghai, Fujian, Guangdong, Hainan, and Tianjin, as well as the northwestern provinces of Qinghai and Xinjiang. Conversely, provinces with lower agricultural carbon emission efficiency are concentrated in the central and western regions, such as Jilin, Anhui, Gansu, and Inner Mongolia. In terms of regional comparison, South China demonstrates the highest agricultural carbon emission efficiency, followed by the Northwest China, Southwest China, East China, North China, Central China and Northeast China. The carbon emission efficiency in South China reached 1.126 in 2021, while Northeast China’s efficiency, which is currently the lowest, stands at 0.417. Furthermore, the southwest region has experienced the most significant improvement in agricultural carbon emission efficiency, with an increase of 0.810 over the past decade. This improvement can largely be attributed to the active promotion of agricultural green projects in Guizhou, which have modernized the agricultural production system and significantly improved the efficiency of agricultural carbon emissions in the region. In contrast, the growth rate in the Northeast region has been slower, with an increase of just 0.157 from 2012 to 2021. Based on these findings, it can be concluded that there is still ample room for improvement in the efficiency of China’s agricultural carbon emissions. Further efforts to reduce carbon emissions in the agriculture and rural sectors are essential.

### 4.2 Decoupling analysis between agricultural carbon emission efficiency and agricultural economy

Based on the research results in formula (3) and Table 4, the decoupling index between China’s agricultural carbon emission efficiency and total agricultural output value is calculated for 2012–2021. The results are shown in Table 5.

**Table 5:** Decoupling results between ACEE and AEG in China, 2012–2021.

Region	Province	2012	2013	2014	2015	2016	2017	2018	2019	2020	2021
North China	Beijing	0.643 (W)	1.516 (E)	0.292 (W)	1.207 (E)	1.069 (D)	0.301 (W)	0.031 (W)	2.930 (E)	-8.269 (S)	4.562 (E)
	Tianjin	1.051 (D)	1.361 (E)	0.605 (W)	2.607 (E)	1.065 (D)	2.230 (E)	2.593 (E)	1.423 (E)	-57.022 (I)	2.988 (E)
	Hebei	1.512 (E)	1.835 (E)	-0.742 (S)	-0.361 (S)	-0.217 (S)	1.179 (W)	2.536 (W)	0.600 (D)	5.556 (E)	0.807 (W)
	Shanxi	1.269 (E)	3.019 (E)	3.075 (E)	0.460 (N)	-1.058 (S)	0.523 (W)	0.574 (W)	0.824 (D)	3.025 (E)	0.538 (W)
	Inner Mongoria	-0.035 (S)	0.935 (D)	0.029 (W)	-0.651 (S)	-0.653 (S)	-0.590 (S)	1.073 (D)	0.890 (D)	38.077 (E)	0.209 (W)
	Mean value	0.827 (D)	1.486 (E)	0.250 (W)	0.447 (W)	0.290 (W)	0.669 (W)	1.159 (D)	1.333 (E)	5.266 (E)	1.460 (E)
Northeast China	Liaoning	1.308 (E)	0.982 (D)	0.064 (W)	17.573 (E)	17.968 (E)	0.454 (W)	1.137 (D)	2.063 (E)	17.157 (E)	0.934 (D)
	Jilin	0.187 (W)	0.538 (W)	-0.235 (S)	-8.739 (S)	-1.105 (S)	-6.885 (S)	4.074 (E)	0.625 (W)	4.751 (E)	0.887 (D)
	Heilongjiang	1.166 (D)	2.832 (E)	2.041 (E)	0.887 (G)	-1.997 (S)	2.586 (E)	1.209 (E)	1.873 (E)	10.248 (E)	0.519 (W)
	Mean value	0.811 (D)	1.408 (E)	0.344 (W)	-2.963 (I)	1.105 (D)	-0.677 (S)	1.763 (E)	1.629 (E)	6.325 (E)	1.320 (E)
East China	Shanghai	-3.689 (S)	0.707 (W)	-0.682 (S)	-0.889 (S)	-0.510 (S)	0.629 (W)	5.082 (E)	-1.552 (S)	-1.670 (S)	1.760 (E)
	Jiangsu	-0.888 (S)	0.573 (W)	0.519 (W)	1.131 (D)	1.310 (E)	0.402 (W)	0.112 (W)	0.807 (D)	2.121 (E)	0.828 (D)
	Zhejiang	-0.270 (S)	1.087 (D)	0.932 (D)	0.574 (W)	0.581 (W)	1.376 (E)	0.556 (W)	11.779 (E)	-10.801 (S)	0.555 (W)
	Anhui	0.349 (W)	0.269 (W)	0.499 (W)	0.229 (W)	0.128 (W)	0.587 (W)	0.007 (W)	0.708 (W)	5.812 (E)	1.042 (D)
	Fujian	1.860 (E)	0.594 (W)	1.101 (D)	0.865 (D)	0.626 (W)	0.484 (W)	0.689 (W)	1.057 (D)	1.917 (E)	1.286 (E)
	Jiangxi	0.672 (W)	1.709 (E)	0.631 (W)	2.062 (E)	1.529 (E)	0.527 (W)	0.754 (W)	1.224 (E)	1.879 (E)	0.424 (W)
	Shandong	-0.079 (S)	1.384 (E)	10.116 (E)	-4.257 (S)	-6.023 (S)	0.463 (W)	1.600 (E)	1.550 (E)	2.386 (E)	1.171 (D)
	Mean value	-0.008 (S)	0.894 (D)	1.625 (E)	-0.089 (S)	-0.074 (S)	0.640 (W)	1.098 (D)	2.387 (E)	0.297 (W)	0.979 (D)
Central China	Henan	1.578 (E)	0.548 (W)	0.826 (D)	0.589 (W)	0.501 (W)	0.610 (W)	1.036 (D)	1.459 (E)	17.117 (E)	1.308 (E)
	Hubei	0.309 (W)	0.423 (W)	0.080 (W)	0.079 (W)	0.059 (W)	0.664 (W)	0.425 (W)	1.476 (E)	-1.610 (I)	0.783 (W)
	Hunan	0.724 (W)	-0.301 (S)	0.168 (W)	-0.006 (S)	-0.008 (S)	0.516 (W)	0.638 (W)	1.655 (E)	4.111 (E)	0.870 (D)
	Mean value	0.808 (D)	0.217 (W)	0.336 (W)	0.192 (W)	0.181 (W)	0.602 (W)	0.695 (W)	1.540 (E)	-2.181 (I)	0.919 (W)
South China	Guangdong	1.592 (E)	1.073 (D)	0.524 (W)	0.604 (W)	0.584 (W)	0.425 (W)	1.301 (E)	1.966 (E)	2.820 (E)	1.469 (E)
	Guangxi	0.478 (W)	0.526 (W)	0.558 (W)	0.437 (W)	0.436 (W)	1.030 (D)	0.795 (W)	1.788 (E)	1.299 (E)	1.126 (D)
	Hainan	1.005 (D)	0.173 (W)	1.116 (D)	0.702 (W)	0.609 (W)	0.373 (W)	1.089 (D)	1.499 (E)	1.934 (E)	6.775 (E)
	Mean value	0.979 (D)	0.562 (W)	0.754 (W)	0.578 (W)	0.546 (W)	0.606 (W)	1.050 (D)	1.746 (E)	1.930 (E)	3.436 (E)
Southwest China	Chongqing	1.179 (D)	0.640 (W)	0.650 (W)	0.414 (W)	0.325 (W)	0.347 (W)	1.433 (E)	1.114 (D)	2.806 (E)	1.110 (D)
	Sichuan	0.962 (D)	0.286 (W)	0.576 (W)	1.238 (E)	0.674 (W)	0.564 (W)	0.371 (W)	0.853 (D)	1.844 (E)	0.942 (D)
	Guizhou	1.199 (D)	0.948 (D)	1.769 (E)	2.012 (E)	2.527 (E)	0.766 (W)	0.982 (D)	1.944 (E)	1.849 (E)	17.548 (E)
	Yunnan	0.906 (D)	0.877 (D)	0.650 (W)	-0.068 (S)	-0.047 (S)	0.338 (W)	1.628 (E)	2.096 (E)	1.613 (E)	1.930 (E)
	Mean value	1.069 (D)	0.736 (W)	1.004 (D)	1.100 (D)	0.928 (D)	0.522 (W)	1.062 (D)	1.552 (E)	2.043 (E)	4.672 (E)
Northwest China	Shaanxi	0.474 (W)	1.011 (D)	0.992 (D)	0.106 (W)	0.047 (W)	0.398 (W)	0.628 (W)	1.335 (E)	20.230 (E)	1.187 (D)
	Gansu	0.912 (D)	0.603 (W)	0.357 (W)	7.720 (E)	0.843 (D)	2.615 (E)	1.270 (E)	1.793 (E)	2.986 (E)	1.762 (E)
	Qinghai	1.173 (D)	1.712 (E)	0.135 (W)	-0.355 (S)	-0.255 (S)	0.406 (W)	0.538 (W)	1.964 (E)	4.933 (E)	3.371 (E)
	Ningxia	0.843 (D)	1.436 (E)	0.938 (D)	3.069 (E)	1.670 (E)	0.254 (W)	1.376 (E)	-0.329 (S)	3.683 (E)	0.220 (W)
	Xinjiang	0.830 (D)	0.033 (W)	-0.797 (S)	9.527 (E)	1.251 (E)	0.359 (W)	0.721 (W)	0.623 (W)	8.961 (E)	2.126 (E)
	Mean value	0.819 (D)	0.921 (D)	0.260 (W)	1.125 (D)	0.541 (W)	0.585 (W)	0.875 (W)	1.115 (D)	5.414 (E)	1.670 (E)
National mean value		0.785 (W)	0.835 (D)	0.715 (W)	0.594 (W)	0.449 (W)	0.516 (W)	1.043 (D)	1.618 (E)	2.728 (E)	2.065 (E)

Table 5 reveals some characteristics of the decoupling relationship between China’s agricultural carbon emission efficiency and agricultural economy from 2012 to 2021. From 2012 to 2018, China experienced weak decoupling (W) between agricultural carbon emission efficiency and agricultural economy. However, from 2019 to 2021, the dominant decoupling characteristic shifted to expansion negative decoupling (E). The decoupling index demonstrated an upward trend, indicating a significant improvement in the decoupling status. In 2012, both agricultural carbon emission efficiency and the agricultural economy were growing, with carbon emission efficiency growing at a faster rate than the agricultural economy. Notably, six provinces exhibited the best state of expansion negative decoupling (E). After 2019, more than half of the provinces in China showcased the ideal state of expansion negative decoupling (E). This transition from weak decoupling (W) to a more stable state of expansion negative decoupling (E) became the general trend. The mean value of the decoupling index for Northwest China shifted from 1.115 (D) in 2019 to 5.414 (E) in 2020, and to 1.670 (E) in 2021. By 2021, no province was in an undesirable state of declining carbon emission efficiency while the agricultural economy was rising. It shows that the Chinese government is paying more attention to the issue of carbon emission efficiency in the process of agricultural production and development, and most regions have achieved significant results in promoting eco-agriculture. However, in 2021, ten provinces still exhibited an expansion connection (D) state and one province exhibited a weak decoupling (W) state, indicating an unstable decoupling state between the agricultural economy and carbon emission efficiency. Therefore, it is necessary to continue exploring production methods and technologies that are low-carbon and low-energy-consuming to improve agricultural carbon emission efficiency.

Secondly, there are significant variations in the decoupling relationship between agricultural carbon emission efficiency and the agricultural economy across different regions. In North China, South China, and Southwest China, there has been an upward trend with expansion negative decoupling for three years. From 2012 to 2017, most areas were in a state of weak decoupling (W), but it had been in the ideal state of expansion negative decoupling (E) since 2018. In Northwest China and Central China, weak decoupling (W) or expansion connection (D) is the main characteristic during the ten-year sample period and the status is relatively stable. The overall decoupling status between agricultural carbon emission efficiency and its economic growth in Northeast and East China has high variability. For example, Northeast China had a decoupling index of -2.963 (I) in 2015, 1.105 (D) in 2016, and -0.677 (S) in 2017. This indicates that maintaining the expansion of the negative decoupling status in Northeast China is more challenging. Thus, more efforts are needed from multiple actors, including governments, businesses, and farmers, to promote the decoupling of agricultural carbon efficiency from the agricultural economy.

In summary, the average decoupling index between agricultural carbon emission efficiency and the agricultural economy in China increased from 0.785 (W) to 2.065 (E). While significant progress has been made in developing low-carbon agriculture, further efforts are needed to strengthen carbon reduction in agriculture, especially to address regional disparities. In North China, South China, Southwest China, and Northwest China, the average decoupling indexes of agricultural carbon emission efficiency and the agricultural economy are positive, indicating better decoupling effects. However, in Northeast China and East China, the average decoupling indexes appear strong decoupling states, with indexes of -0.677 (S) in 2017 and -0.074 (S) in 2016, respectively. Future agricultural carbon emission reduction policies should establish a horizontal carbon compensation mechanism, taking into full consideration inter-regional differences and spatial correlation characteristics, aiming to break the constraints of administrative boundaries.

## 5 Research conclusions and discussion

### 5.1 Conclusions

This study employs the Super-SBM model to measure the agricultural carbon emission efficiency of 30 provinces in China from 2012 to 2021. Additionally, the Tapio decoupling model is applied to analyze the decoupling relationship between agricultural carbon emission efficiency and the agricultural economy. The study finds: Firstly, the average agricultural carbon emission efficiency of the 30 provinces in China significantly increased, with the average value rising from 0.349 in 2012 to 0.807 in 2021. Secondly, there are evident disparities in agricultural carbon emission efficiency among different regions. The highest efficiency is observed in South China, followed by Northwest China, Southwest China, East China, North China, Central China and Northeast China. Thirdly, from 2012 to 2021, the decoupling status between China’s agricultural carbon emission efficiency and the agricultural economy shifted from mainly weak decoupling to predominantly expanding negative decoupling. In 2019, 18 provinces achieved the ideal state of expanding negative decoupling. However, the decoupling status remains unstable, indicating that there is still room for improvement in decoupling agricultural carbon emission efficiency from agricultural economy.

Based on the research findings, several recommendations are proposed for promoting the development of low-carbon agriculture.

Firstly, attention should be paid to the regional disparities in agricultural carbon emission efficiency, and a coordinated development mechanism for different agricultural regions should be established. The research indicates significant regional disparities in China’s agricultural carbon emission efficiency. The government could implement coordinated emission reduction actions with neighboring provinces and organize activities such as technical assistance, counterpart support, and experience exchanges. Secondly, differentiated emission reduction and carbon sequestration strategies should be formulated based on the distinct characteristics of various agricultural regions. Each region should propose carbon emissions reduction plans that align with their specific natural resources, climatic conditions, and other factors. For example, the Northeast region should focus on protecting black soil and promoting conservation tillage techniques. The Central China region should aim to improve resource utilization efficiency, standardize and rectify energy-intensive agricultural practices, and prioritize the promotion of ecological circular agriculture. The North China, East China, and Southwest China regions should leverage their regional advantages, guiding agricultural market entities to adopt foreign low-carbon technologies and attract capital investment. The Northwest region should capitalize on its unique geography and climate to cultivate special agricultural products and increase agricultural economic output. Thirdly, each region should rationally plan agricultural production input factors and optimize agricultural resource allocation. China’s agricultural carbon emission efficiency remains in a state of fluctuation, necessitating innovation in the planning and allocation of agricultural production factors. It is essential to develop vigorously new agricultural technologies, optimize the agricultural planting structure, and cultivate superior crop varieties with characteristics such as drought resistance, water-saving and pest resistance.

### 5.2 Discussion

Agriculture is crucial to human survival. However, greenhouse gas caused by agriculture is one of the major sources of global greenhouse gas emissions. Therefore, it is necessary to promote increased agricultural production and income while reducing agricultural carbon emissions. Currently, most scholars focus on studying agricultural carbon emission efficiency and its influencing factors, but they do not analyze the decoupling relationship between agricultural carbon emission efficiency and the agricultural economy. The contribution of this paper is to analyze this relationship using the Tapio decoupling model. The study’s findings can provide insights for the development of low-carbon agriculture, however, it still has some limitations. Firstly, the panel data collected only cover 30 provinces in China, future studies should refine the analysis to include Chinese prefecture-level cities and counties. Secondly, this research focuses solely on China, future studies should explore the agricultural carbon emission efficiency of other countries and conduct comparative studies with China’s agricultural carbon emission efficiency.

## Supporting information

S1 Data (XLSX)
